# Investigation of APOE4's impact on cholinergic signaling

**DOI:** 10.1002/alz70855_098606

**Published:** 2025-12-23

**Authors:** Shreyan Kancharla, Pattie Lamb, Jerrel Yakel, Qing Cheng

**Affiliations:** ^1^ North Carolina Central University, Durham, NC, USA; ^2^ National Institute of Environmental Health Sciences, Durham, NC, USA

## Abstract

**Background:**

Sporadic Alzheimer's disease (AD) is a progressive neurodegenerative disease with a complex and poorly understood etiology. While amyloid‐beta and phosphorylated tau are prominent in AD pathogenesis, deficits in cholinergic signaling play a critical role in cognitive impairments and early clinical symptoms of AD. This project investigates the impact of APOE4, the strongest genetic risk factor for sporadic AD, on cholinergic signaling in the brain.

**Method:**

Brain samples were collected from 12‐month‐old female APOE3 and APOE4 knock‐in transgenic mice. Neuronal counts in the hippocampus were assessed using Nissl staining, while cholinergic neuron counts and intracellular amyloid‐beta levels were determined by immunohistochemistry. The expression of α7 nicotinic acetylcholine receptors (α7 nAChR) was quantified using RNAscope multiplex assays. All images were captured with a Zeiss LSM 800 confocal microscope.

**Result:**

The number of cholinergic neurons in the septum was significantly reduced in 12‐month‐old female APOE4 mice compared to APOE3 controls. Additionally, APOE4 female mice exhibited substantial neuronal loss across all hippocampal regions, including the dentate gyrus, CA3, and CA1. Our findings revealed a pronounced decrease in α7 nAChR mRNA expression in APOE4 dentate granule cells, hilar GABAergic interneurons, and CA3 pyramidal neurons, with minimal changes observed in CA1 neurons. Moreover, intracellular amyloid‐beta levels were elevated in the dentate neurons of both APOE4 and α7 nAChR knockout mice.

**Conclusion:**

APOE4 mice exhibit impaired cholinergic signaling, which may contribute to disinhibition and hyperexcitability of glutamatergic neurons, potentially exacerbating AD pathogenesis.

